# Epidemiology and Patterns of Pediatric Visits and Referrals in a Model Primary Health Care Centre in Saudi Arabia: A Retrospective Chart Review Study

**DOI:** 10.3390/healthcare13233005

**Published:** 2025-11-21

**Authors:** Reem S. AlOmar, Nouf A. AlShamlan, Abdulrahman A. Al-Abdulazeem, Ahmed M. Al-Turki, Ahmed A. Al Yateem, Reema J. Alghamdi, Najla A. Alhamed, Sameerah Motabgani, Assim M. AlAbdulKader, Wejdan M. Al-Johani, Malak A. Al Shammari

**Affiliations:** 1 Department of Family and Community Medicine, College of Medicine, Imam Abdulrahman Bin Faisal University, Dammam 31441, Saudi Arabia; 2 College of Medicine, Imam Abdulrahman Bin Faisal University, Dammam 31441, Saudi Arabia; 3 Pharmaceutical Affairs, King Fahad Specialist Hospital, Eastern Health Cluster, Dammam 32253, Saudi Arabia

**Keywords:** epidemiology, pediatric visits, primary healthcare, public health, referrals

## Abstract

**Background**: Primary healthcare plays a vital role in delivering pediatric services. This study aimed to examine the epidemiology of pediatric visits to a model primary healthcare (PHC) center and identify factors associated with referrals to specialized care. **Methods**: A retrospective chart review was conducted for all pediatric visits between January and December 2024 at a model PHC center affiliated with an academic medical city in the Kingdom of Saudi Arabia (KSA). Descriptive statistics, chi-squared tests, and multivariable logistic regression were used to assess predictors of referral. Diagnoses were categorized, and clinic types stratified to explore seasonal and diagnostic trends. Ethical approval was obtained prior to data access. **Results**: A total of 4520 pediatric visits were analyzed. Just over half of the patients were female, and the largest age group was school-aged children (38.1%). Visit frequency peaked in winter and spring. Most visits (78.4%) were first-time consultations, and the majority occurred in general family medicine clinics. Overall, 10.95% of visits resulted in referrals. Referrals were more common during outpatient consultations than urgent care visits and were strongly associated with specific diagnoses, particularly neurological (aOR = 11.73), eye (aOR = 8.77), ENT-related conditions (aOR = 7.73), and genitourinary or pubertal conditions (aOR = 6.60). Demographic variables such as sex and nationality were not significant predictors. **Conclusions**: The observed referral rate may indicate effective gatekeeping within Saudi primary care, though referral frequency alone cannot determine appropriateness. Enhancing diagnostic support and behavioral health integration could further optimize referral practices and support Vision 2030 goals for strengthened child health services.

## 1. Introduction

Primary healthcare is integral in the provision of pediatric services worldwide, offering a first point of contact for the diagnosis, prevention, and management of acute and chronic childhood conditions. International studies have emphasized how understanding the volume, timing, and nature of pediatric visits can inform health service planning, reduce unnecessary referrals, and strengthen the continuity of care [1,2].

Globally, referral rates from primary care to specialist services vary widely, ranging from 5% to 20%, influenced by healthcare system structure, availability of services, gatekeeping models, and physician training levels [3,4]. In high-income countries with strong primary care systems such as the UK and the Netherlands, referral rates are typically lower due to emphasis on comprehensive care and continuity within general practice [5]. Whereas in settings where primary healthcare (PHC) centers have limited diagnostic capacity or where health-seeking behavior favors direct specialist access, higher referral rates have been documented, particularly in parts of Asia and the Middle East [6]. In the context of the Kingdom of Saudi Arabia (KSA), studies have noted substantial variation in referral patterns between institutions and regions. For example, one study found that the referral rate was 16% to a wide variety of clinics, while other studies focusing on specific services, such as ENT, have documented more selective referral rates from family medicine clinics, particularly where multidisciplinary care or specialist clinics are embedded within the PHC centers [7,8]. These variabilities reflect both the evolving role of PHC centers in the healthcare system and the need to better understand how patient, provider, and system-level factors shape referral decisions.

In the KSA, PHC centers act as gatekeepers to specialized services under the Ministry of Health’s national referral policy. According to this framework, patients are referred to higher levels of care only when their clinical needs exceed the diagnostic or therapeutic capacity of the PHC center. Referrals are guided by standardized clinical criteria, electronic referral systems, and coordination protocols between PHC centers and specialized hospitals, ensuring continuity, efficiency, and equity of access. This gatekeeping model is central to the ongoing health transformation initiatives under Vision 2030 [9]. Model PHC centers which are defined as exemplary centers with multidisciplinary team-based care, and comprehensive family medicine, preventive, and rehabilitative clinics are central to this strategy [10]. They are intended to function as benchmarks of integrated care and early intervention, incorporating urgent care access, and digitally enabled referral systems.

Several studies in the Saudi context have addressed referral volume to a specialist care clinic or within emergency departments. However, detailed epidemiologic evaluations of pediatric visit patterns within a model PHC center, including seasonal distributions, reasons for visit, and predictors of referral remain scarce [7,11]. From an epidemiological standpoint, analyzing patterns of pediatric healthcare utilization and referrals provides critical insights into disease burden, service accessibility and the performance of systems at the population level [12]. Therefore, this study presents a year-long epidemiological analysis of all pediatric visits to a model PHC center in the KSA. The objectives are two-fold. First, to explore the epidemiology and patterns of pediatric patients’ visits in terms of temporality and characteristics of these visits and second, to examine the predictors of referrals to specialist care.

## 2. Materials and Methods

### 2.1. Study Design and Setting

This retrospective chart review study was conducted at a Family and Community Medicine Centre which is a part of an academic medical city in the KSA. The center offers a broad range of services including general family medicine clinics treating patients across all age groups, well-baby and child health clinics, women’s health services, and a robust preventive care program encompassing premarital counselling, Hajj and travel medicine. Furthermore, an urgent care track is also available to manage cases that need fast access to care, supported by a well-equipped treatment and observation unit composed of 6 beds. Additionally, a selection of specialty clinics, including dermatology, ENT, ophthalmology, orthopedics, and neurology operate on a weekly basis, further enhancing access to integrated care. This multifaceted structure positions the center as a model PHC in both service provision and the academic advancement of family medicine in the region.

### 2.2. Ethical Considerations

The study was ethically approved by the Institutional Review Board of Imam Abdulrahman Bin Faisal University (IRB-2024-01-611). All data were treated with strict confidentiality and used solely for research purposes. The study was conducted in accordance with the principles outlined in the Declaration of Helsinki.

### 2.3. Study Participants

In this PHC center, pediatric visits are defined operationally as those involving patients under 14 years of age, since adolescents aged 14 years and above are routinely seen in adult clinics. Therefore, the study included all pediatric patients visits aged <14 years of age to any of the clinics at the center between the 1 January 2024 to the 31 December 2024.

### 2.4. Data Collection Tool

Data was obtained after ethics approval from the electronically stored patients’ files. A data collection sheet was designed after reviewing studies with similar objectives [11,13]. The age groups included infants (<1 year old), toddlers (1 year < 3 years), preschoolers (3 years < 6 years), schoolers (6 years < 12 years) and adolescents (12 years < 14 years). Other demographic variables included sex and nationality. Also, the data collection sheet included visit specific characteristics such as the month of the visit, type of clinic, type of visit, whether the patient had been referred or not, and if referred, the name of the recipient department and finally the primary diagnosis. Diagnoses were extracted according to the International Classification of Diseases, 10th revision (ICD-10) codes entered by treating physicians in the electronic medical record system. These codes were reviewed and recategorized into broader diagnostic categories (e.g., respiratory, ENT, gastrointestinal, dermatologic, allergic/skin, musculoskeletal, genitourinary, developmental-psychiatric-behavioral) by two consultants in family medicine and one epidemiologist. When multiple diagnoses were recorded, the primary diagnosis corresponding to the main reason for visit was retained. All reviewers independently verified the categorization and resolved any discrepancies by consensus. The data was collected between February and June 2025.

### 2.5. Statistical Analysis

All categorical variables were described as frequencies and percentages. Bivariate associations were performed through Chi-squared tests of associations. A *p*-value of <0.05 was considered statistically significant. The outcome of the study was whether a patient was referred to a specialist clinic. Unadjusted and adjusted binary logistic regression models were used to compute Odds Ratios (ORs) and their 95% Confidence Intervals (CIs). Routine check-up visits were used as the reference category for diagnosis because they represent non-illness encounters where referral is least anticipated, allowing other diagnostic categories to be compared against a clinically neutral baseline. Model assumptions and fit were assessed through post-estimation diagnostics, including checks for multicollinearity using Variance Inflation Factors (VIF < 5), model discrimination using the C-statistic, calibration using the Hosmer–Lemeshow goodness-of-fit test, and specification using the link test. All analyses were performed in STATA software version 15.0 (Stata Corporation, College Station, TX, USA) [14].

## 3. Results

### 3.1. Overall Characteristics of Pediatric Visits and Temporal Trends

Overall sociodemographic characteristics are presented in Table 1. A total of 4520 pediatric visits were recorded in 2024. Of these, 48.78% were male and 51.22% were female. The most common age group was for schoolers comprising 38.08% of the total pediatric population, followed by preschoolers (18.92%). Most visits were for Saudi nationals (71.62%) (Table 1).

Table 2 presents the characteristics of the visits. Of the total visits, 10.95% resulted in a referral. With regard to the type of clinic, 77.52% of the visits were to the outpatient primary care setting, whereas 22.48% were to the urgent care setting. Similarly, most of the visits were first consultations, compared to only 21.59% for follow-up. Most visits were completed by both the doctor and the patient and were stated as departed (78.14%), whereas 15.64% of the patients did not attend their appointment. Visits were similarly distributed across seasons but were slightly higher for winter and spring than for autumn and summer. With regard to the primary diagnosis, the most registered was for routine check-ups (33.61%), followed by preventive services and vaccinations in 17.68% and respiratory infections in 12.12% of visits. The least common registered diagnosis was neurological disorders.

**Figure 1:**
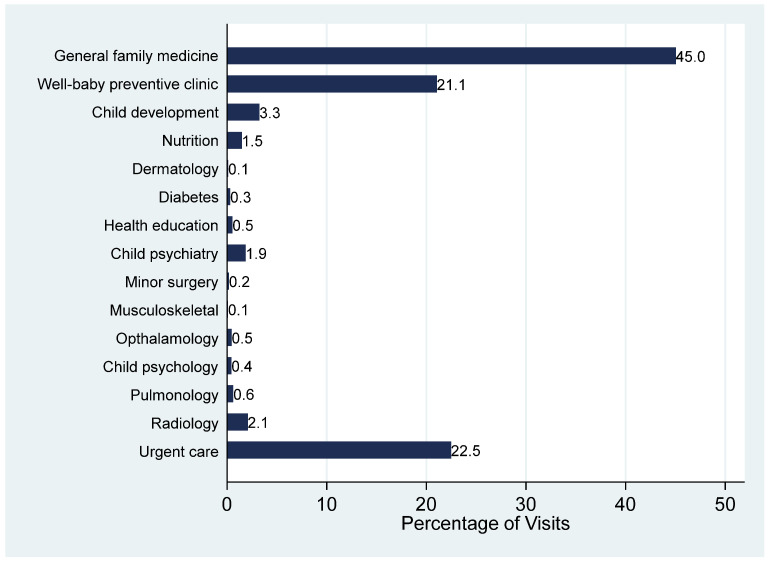
Pattern of pediatric visits according to the type of primary care clinic.

Figure 1 displays the distribution of pediatric visits across all clinic types during the study period. General family medicine clinics accounted for the largest share of visits, comprising 45.0% of all encounters. Urgent care services followed at 22.5% and well-baby and preventive care clinics followed at 23%. Other outpatient clinics, including child development, nutrition, child psychiatry, radiology, and diabetes clinics, contributed smaller proportions individually, each representing less than 5% of total visits.

**Figure 2:**
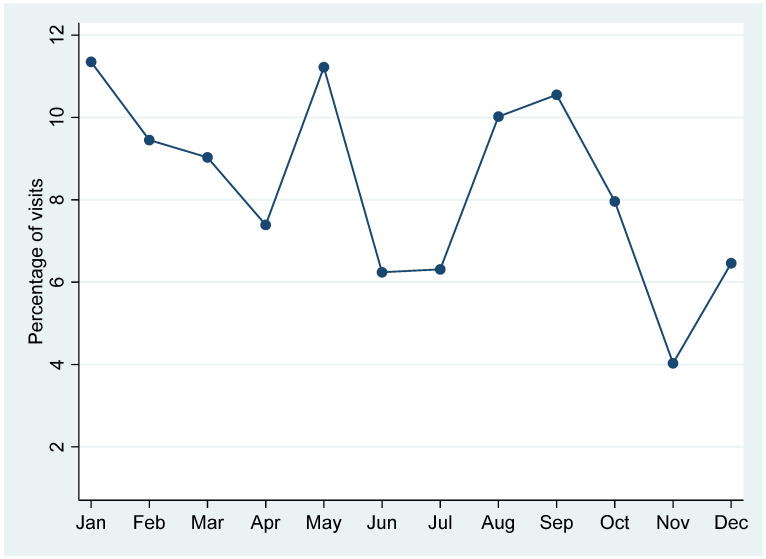
Pattern of pediatric visits across months for all types of primary care clinics.

Figure 2 illustrates the temporal distribution of pediatric visits by months. Monthly trends show that the highest volumes occurred in January (11.6%), May (11.5%) and September (10.4%), indicating a winter-to-early-spring peak. In contrast, November had the least number of visits throughout the year (4.1%).

### 3.2. Characteristics of Pediatric Visits According to Referral Type

Table 3 shows the distribution of sociodemographic characteristics of pediatric visits according to referrals. No statistically significant difference in sex of patients was observed in terms of referral, nor for nationality. However, for age, a highly statistically significant difference was observed (*p* < 0.001), where schoolers were observed to have a higher proportion of referrals.

Table 4 presents the distribution of visit characteristics stratified by referral status. Referrals were significantly more frequent among patients seen in the outpatient primary care setting compared to urgent care (13.0% and 3.7%, respectively) (*p* < 0.001). The proportion of referred cases did not differ significantly by type of visit. Similarly, no significant seasonal variation was observed in referral distribution. However, substantial differences emerged when stratifying by primary diagnosis (*p* < 0.001). The highest referral proportions were observed among patients diagnosed with neurological disorders (36.0%), eye conditions (28.95%), genitourinary and puberty-related issues (28.13%), and musculoskeletal or orthopedic conditions (25.34%). In contrast, referrals were less common among cases involving respiratory infections (4.38%), preventive services and vaccinations (3.88%), and routine check-ups (6.19%).

**Figure 3:**
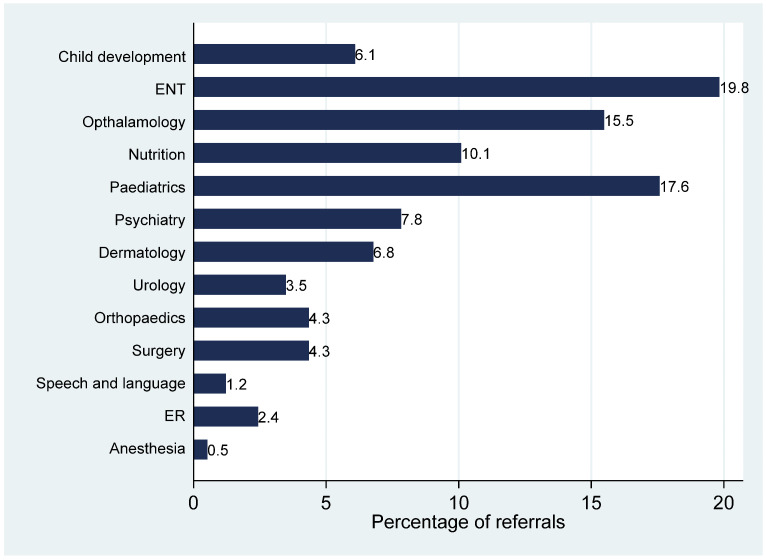
Percentages of referrals by receiving departments.

Figure 3 illustrates the percentage distribution of referrals by receiving department. The most frequently referred-to services were ENT and pediatrics, accounting for approximately 19% and 17% of all referrals, respectively. Ophthalmology followed closely, receiving around 15.5% of referrals, while nutrition services received approximately 10%. Mental health–related services were also notable, with psychiatry and child development each receiving just over 7% of referrals. Less commonly referred departments included speech and language, emergency services, and anesthesia, all representing under 3% of total referrals.

### 3.3. Predictors of Primary Care Pediatric Visit Referrals

Table 5 displays the results of the multivariable logistic regression model identifying predictors of referral. Post-estimation diagnostics confirmed that the model met all assumptions and demonstrated good performance. Multicollinearity was not detected, with all VIF values below 2. The model showed acceptable discrimination (C-statistic = 0.78), indicating adequate ability to rank visits according to their predicted probability of referral. Calibration was satisfactory based on the Hosmer–Lemeshow goodness-of-fit test (*p* = 0.41), and the link test indicated correct model specification with a non-significant squared predicted-value term.

After adjustment, sex was not significantly associated with referral status. However, adolescents were found to have a statistically significantly lower odds of referral (Adjusted OR = 0.69, 95% CI = 0.51–0.93).

With regard to the type of clinic, patients seen in urgent care settings had a significantly lower odds of referral compared to those seen in outpatient clinics (adjusted OR = 0.17, 95% CI = 0.12–0.26). Similarly, the type of visit was also a significant predictor for referrals, where patients attending follow-up visits had lower odds of referrals (adjusted OR = 0.78, 95% CI = 0.61–0.99).

When stratified by primary diagnosis, several categories were significantly associated with increased likelihood of referral, using routine check-ups as the reference group. The highest adjusted odds were observed among children presenting with neurological conditions (adjusted OR = 11.73, 95% CI = 4.83–28.47), eye conditions (adjusted OR = 8.77, 95% CI = 4.96–15.51), ENT related condition (adjusted OR = 7.73, 95% CI = 4.26–14.04), genitourinary or pubertal concerns (adjusted OR = 6.60, 95% CI = 3.97–10.97), and musculoskeletal or orthopedic diagnoses (adjusted OR = 6.57, 95% CI = 4.20–10.27). Referrals were also more likely for allergic and skin conditions, endocrine-related conditions and developmental, psychiatric and behavioral conditions. Conversely, preventive services were significantly less likely to result in referral compared to routine visits (adjusted OR = 0.45, 95% CI: 0.29–0.71).

## 4. Discussion

This study offers a comprehensive overview of pediatric healthcare utilization and referral patterns within a family medicine setting in Saudi Arabia. By analyzing over 4500 pediatric visits across an entire year, the findings shed light on the demographic and clinical characteristics of children accessing primary care, the seasonal trends in visit frequency, and the predictors of referrals to other specialties. Understanding these patterns is essential for optimizing the gatekeeping role of family physicians, enhancing the efficiency of referral pathways, and aligning pediatric primary care delivery with the goals of the KSA’s Vision 2030 health sector transformation [9]. The results highlight both strengths and opportunities for improvement in managing pediatric conditions within primary care and ensuring timely, appropriate access to specialized services.

With regard to referrals, only 10.95% of visits were referred for further evaluation and management to a specialized clinic. This finding suggests that most pediatric cases were effectively managed within the initial care setting, possibly reflecting diagnostic confidence among primary care providers or limited perceived need for specialist input. However, referral rate alone should be interpreted with caution, as a lower proportion could also reflect potential under-referral or differing thresholds for specialist involvement. In comparison, a study conducted in Australia reported that just over half of pediatric specialist referrals originated from general practitioners, reflecting a healthcare system with more direct access to specialist care and less structured gatekeeping [2]. These contextual differences in healthcare organization, access pathways, and referral regulations likely account for the observed variation between settings.

In the present study, school-aged children comprised the most frequent age group presented to the family medicine clinics and were the highest group referred to other clinics, which is consistent with prior studies indicating that this population presents more often for immunizations, vision or hearing screening, and assessments related to learning or behavioral issues [15,16]. Moreover, the apparent association between age and referral observed in bivariate analysis diminished after adjustment for these clinical and visit characteristics, suggesting confounding. Younger school-age children were more likely to attend for acute illnesses requiring review, whereas adolescents were more often presented for preventive or behavioral issues managed within primary care. After accounting for these factors, only adolescents remained less likely to be referred, possibly reflecting a lower clinical need for specialist involvement in this group [17]. Furthermore, providing counselling and approaching adolescent patients are important competencies for family medicine residents that were mentioned clearly in the Saudi Board for Family Medicine curriculum [12].

Interestingly, analysis revealed that neither nationality nor sex had significant associations with referral rates, underscoring that clinical presentation and diagnostic category are stronger referral drivers than sociodemographic characteristics. This suggests that family physicians in this setting make referral decisions based on medical necessities rather than patient demographics, supporting equity in care delivery.

Seasonal variation was observed mainly in the volume of pediatric visits, with peaks during winter and spring months, consistent with higher incidence of respiratory infections and school-entry examinations. However, referral rates did not differ significantly across seasons, suggesting that these fluctuations primarily reflect visit frequency rather than referral outcomes [11,18,19].

With regard to the types of clinics, the findings of this study underscore the pivotal role of general family medicine clinics in the delivery of pediatric healthcare, with nearly half of all pediatric visits occurring in this setting. Furthermore, the significant proportion of visits to well-baby and preventive care clinics further emphasizes the healthcare system’s focus on early child development, immunizations, and routine health maintenance. This finding supports the overall government’s healthcare transformation program in promoting preventive services and promoting long-term pediatric health outcomes [9]. Urgent care services were also considerably high, which represents a critical component in addressing immediate and unscheduled health concerns. It reflects both the accessibility of these services and parental preferences for timely care outside of traditional clinic scheduled appointments. Research shows that while primary care visits may be decreasing, urgent care utilization is increasing, particularly among those who often seek care across multiple settings rather than solely relying on their primary care physician, which shows a need to investigate health-seeking behavior [20].

Specialized outpatient services, including child development, nutrition, and diabetes care, collectively contributed to a relatively small fraction of visits. While this may reflect the more focused and referral-based nature of these services, it also highlights the need to ensure that children requiring specialized care are appropriately identified and referred. This finding warrants further investigation into potential barriers, such as limited availability, referral delays, or lack of awareness among caregivers and primary care providers [4].

The data shows that the proportion of follow-up visits was 21.59% overall. This may be due to challenges in continuity of care or limited scheduling capacity for follow-ups, which could have downstream implications on disease monitoring and preventive care adherence. Alternatively, it might suggest that many pediatric concerns are acute or episodic in nature, requiring only a single consultation which is further supported by findings from a previous study, which identified acute fever and upper respiratory tract symptoms as the most common reasons for pediatric emergency department visits, reflecting the predominance of acute presentations in pediatric care [2,11].

The fact that after adjustment, the referral patterns show that urgent care visits had lower odds of referrals is a further indication that urgent care clinics mainly manage acute, self-limiting conditions that can be treated immediately within primary care, while outpatient clinics more often address chronic, developmental, or behavioral issues that may require specialist input. This distinction explains the higher referral frequency in the outpatient setting despite the acute nature of urgent care presentations. Furthermore, the lower odds of referrals for follow-up visits when compared to first consultations indicate that continued care may lessen the need for a specialist evaluation. They also align with prior research showing that medical residency training in family and community medicine significantly reduced referrals from primary to specialized care, an effect that remained even after accounting for potential confounders [21].

With regard to primary diagnoses, their distribution highlights the key role of preventive care and routine assessments in community-based pediatric practice. Where routine check-ups, preventive services and vaccinations and respiratory infections were the most common reasons for visits consecutively. This supports international evidence showing that anticipatory guidance, immunizations, and managing common childhood illnesses are the most frequent reasons for pediatric visits in primary care settings [22,23]. These findings emphasize the importance of maintaining well-child programs and optimizing access to comprehensive preventive services in primary care. When stratified by referral status, the likelihood of referral varied substantially by diagnosis. Although most visits were managed without referral, certain conditions were more often referred. For example, neurological complaints had the highest proportion of referrals, followed by eye conditions, genitourinary or puberty-related issues, and musculoskeletal or orthopedic problems. These findings align with previous studies identifying these categories as common reasons for referral due to their diagnostic complexity and the need for specialized evaluation or imaging [24,25]. Conversely, referrals were lowest for respiratory infections, preventive services, and routine check-ups, indicating proper primary care management of self-limited or protocol-driven conditions. These findings remained in the logistic regression after adjustment, which gives further credence to these results.

Upon analyzing the patterns of referrals among the recipient departments, ENT and general pediatrics accounted for most referrals in the current study, followed by ophthalmology, nutrition, psychiatry, and child development. These findings mirror global trends that identify ENT and vision-related conditions as leading causes of pediatric referrals from primary care, often due to their frequency, chronicity, the need for procedural intervention, the need for enhanced training in primary care, better access to basic diagnostic tools, and implementation of standardized referral guidelines to optimize specialist utilization and improve patient care [7,26,27]. The prominence of referrals to psychiatry and child development clinics in this study is noteworthy, particularly considering growing awareness of pediatric mental health and neurodevelopmental conditions in primary care. These trends call for expanded access to behavioral health services and greater integration of mental health care into primary settings [28].

Interestingly, referrals to emergency services were among the least commonly consulted specialties in the current study. This finding may reflect the competency of family physicians in managing a wide spectrum of acute and urgent pediatric conditions within the primary care setting [12]. Moreover, the relatively low reliance on ER referrals suggests appropriate triaging and effective acute care delivery in the primary care context. Additionally, the presence of embedded urgent care services within family medicine centers may have further reduced the need for emergency referrals by offering same-day care for less severe presentations. These observations underscore the crucial role of family physicians in reducing unnecessary burden on emergency departments and highlight the importance of reinforcing primary care systems to serve as the frontline for pediatric acute care [11].

This study offers several strengths. It is one of the few epidemiological investigations in the KSA to examine pediatric visit patterns and referral predictors within a model PHC setting over an entire calendar year. The setting, which is a digitally enabled academic model PHC center with integrated urgent and specialty clinics, allowed for the inclusion of diverse visit types and clinical encounters. Moreover, the use of routinely collected electronic health data improves accuracy and reduces recall bias. Importantly, the study explored both descriptive trends and multivariable predictors of referral, enabling actionable insights into healthcare delivery and system performance. However, some limitations must be acknowledged. First, being a single-center study, the results may not be fully generalizable to other primary healthcare settings. As data were obtained from electronic medical records, documentation or coding inaccuracies cannot be entirely excluded. The study did not assess the appropriateness or completion of referrals, patient outcomes, or satisfaction, all of which are important for evaluating the effectiveness of the referral process. Additionally, the model PHC may not represent the case mix or operational characteristics of typical primary care centers. Furthermore, the operational definition of pediatric patients (<14 years) reflects the clinic’s service model rather than the broader World Health Organization’s definition; therefore, generalizability to older adolescents may be limited. Future multicenter studies incorporating referral appropriateness, completion rates, and patient-reported outcomes are recommended to build upon these findings.

## 5. Conclusions

This study provides an overview of pediatric healthcare utilization and referral patterns within a model primary healthcare centre in Saudi Arabia. The findings highlight the central role of family medicine clinics in managing pediatric conditions, with relatively few visits resulting in referrals. Referral decisions appeared mainly influenced by clinical factors such as diagnosis, clinic type, and visit type rather than sociodemographic characteristics, reflecting a medically oriented decision process. However, referral frequency alone cannot determine efficiency or appropriateness and should be interpreted within the broader context of healthcare access and system design. These results carry practical implications for health system optimization. From a practice perspective, strengthening diagnostic support, improving access to behavioral and developmental health services, and enhancing communication between primary and specialist care could help optimize referral decisions. Structured feedback from specialists and continuous professional development for PHC physicians may also support appropriate referral practices. These strategies support Vision 2030 objectives to enhance continuity, reduce unnecessary referrals, and improve child health outcomes through a more integrated primary care system.

## Figures and Tables

**Table 1:** Sociodemographic characteristics of pediatric visitors of urgent care clinics in 2024.

Characteristics	N (%) 4520 (100.00)
**Sex**	
Males	2205 (48.78)
Females	2315 (51.22)
**Age group**	
Infants	428 (09.47)
Toddlers	852 (18.85)
Pre-schoolers	855 (18.92)
Schoolers	1721 (38.07)
Adolescents	664 (14.69)
**Nationality**	
Saudi	3237 (71.62)
Non-Saudi	1283 (28.38)

**Table 2:** Characteristics of pediatric visits to urgent care clinics in 2024.

Characteristics	N (%) 4520 (100.00)
**Referrals**	
Referred	495 (10.95)
Not referred	4025 (89.05)
**Type of clinic**	
Outpatient primary care setting	3504 (77.52)
Urgent care	1016 (22.48)
**Type of visit**	
First consultation	3544 (78.41)
Follow-up	976 (21.59)
**Appointment status**	
Departed	3532 (78.14)
Not attended	707 (15.64)
Cancelled	277 (06.13)
Arrived not seen	3 (00.07)
Postponed	1 (00.02)
**Season**	
Winter	1232 (27.26)
Spring	1249 (27.63)
Summer	1020 (22.57)
Autumn	1019 (22.54)
**Primary diagnosis**	
Respiratory infections	548 (12.12)
ENT	79 (01.75)
Allergic and skin conditions	313 (06.93)
Gastrointestinal	128 (02.83)
Nutritional deficiencies	264 (05.84)
Neurological disorders	25 (0.55)
Developmental, psychiatric and behavioral	303 (06.70)
Genitourinary and puberty related	96 (02.12)
Endocrine disorders and diabetes	107 (02.37)
Musculoskeletal and orthopedics	146 (03.23)
Eye conditions	76 (01.68)
Preventive services and vaccinations	799 (17.68)
Unspecified (e.g., general fatigue)	117 (02.59)
Routine check-up	1519 (33.61)

**Table 3:** Sociodemographic characteristics of primary care pediatrics’ visits according to referrals in 2024.

Characteristics	Referrals		*p*-Value
	No N (%) 4025 (89.05)	Yes N (%) 495 (10.95)	
**Sex**			0.34
Males	1954 (88.62)	251 (11.38)	
Females	2071 (89.46)	244 (10.54)	
**Age group**			<0.001
Infants	395 (92.29)	33 (07.71)	
Toddlers	788 (92.49)	64 (07.51)	
Pre-schoolers	751 (87.84)	104 (12.16)	
Schoolers	1504 (87.39)	217 (12.61)	
Adolescents	587 (88.40)	77 (11.60)	
**Nationality**			0.95
Saudi	2882 (89.03)	355 (10.97)	
Non-Saudi	1143 (89.09)	140 (10.91)	

**Table 4:** Primary care pediatric patients’ visit characteristics in 2024 according to referral.

Characteristics	Referrals		*p*-Value
	No N (%) 4025 (89.05)	Yes N (%) 495 (10.95)	
**Type of clinic**			<0.001
Outpatient primary care setting	3047 (86.96)	457 (13.04)	
Urgent care	978 (96.26)	38 (03.74)	
**Type of visit**			0.34
First consultation	3164 (89.28)	380 (10.72)	
Follow-up	861 (88.22)	115 (11.78)	
**Season**			0.48
Winter	1096 (88.96)	136 (11.04)	
Spring	1118 (89.51)	131 (10.49)	
Summer	916 (89.80)	104 (10.20)	
Autumn	895 (87.83)	124 (12.17)	
**Primary diagnosis**			<0.001
Respiratory infections	524 (95.62)	24 (04.38)	
ENT	60 (75.95)	19 (24.05)	
Allergic and skin conditions	233 (74.44)	80 (25.56)	
Gastrointestinal	116 (90.62)	12 (09.38)	
Nutritional deficiencies	224 (84.85)	40 (15.15)	
Neurological disorders	16 (64.00)	9 (36.00)	
Developmental, psychiatric and behavioral	237 (78.22)	66 (21.78)	
Genitourinary and puberty related	69 (71.87)	27 (28.13)	
Endocrine disorders and diabetes	83 (77.57)	24 (22.43)	
Musculoskeletal and orthopedics	109 (74.66)	37 (25.34)	
Eye conditions	54 (71.05)	22 (28.95)	
Preventive services and vaccinations	768 (96.12)	31 (03.88)	
Unspecified (e.g., general fatigue)	107 (91.45)	10 (08.55)	
Routine check-up	1425 (93.81)	94 (06.19)	

**Table 5:** Predictors of referrals according to primary care pediatric patients’ sociodemographic and visit characteristics in 2024.

Characteristic	Odds of Referrals			
	*p*-Value	Unadjusted Odds (95% CI)	*p*-Value	Adjusted Odds (95% CI)
**Sex**				
Males	0.36	1.09 (0.90–1.31)	0.64	1.04 (0.85–1.28)
Females	Ref			
**Age group**				
Infants	0.005	0.57 (0.39–0.84)	0.38	1.21 (0.78–1.87)
Toddlers	<0.001	0.56 (0.42–0.75)	0.71	0.94 (0.67–1.30)
Pre-schoolers	0.74	0.95 (0.74–1.23)	0.80	1.03 (0.79–1.35)
Schoolers	Ref			
Adolescents	0.50	0.90 (0.68–1.19)	0.01	0.69 (0.51–0.93)
**Type of clinic**				
Outpatient primary care setting	Ref			
Urgent care	<0.001	0.25 (0.18–0.36)	<0.001	0.17 (0.12–0.26)
**Type of visit**				
First consultation	Ref			
Follow-up	0.34	1.11 (0.89–1.38)	0.04	0.78 (0.61–0.99)
**Primary diagnosis**				
Respiratory infections	0.12	0.69 (0.43–1.09)	0.14	1.43 (0.88–2.32)
ENT	<0.001	4.80 (2.75–8.37)	<0.001	7.73 (4.26–14.04)
Allergic and skin conditions	<0.001	5.20 (3.74–7.23)	<0.001	5.70 (4.07–8.00)
Gastrointestinal	0.16	1.56 (0.83–2.94)	0.03	2.02 (1.06–3.86)
Nutritional deficiencies	<0.001	2.70 (1.82–4.02)	<0.001	2.52 (1.68–3.77)
Neurological disorders	<0.001	8.52 (3.67–19.80)	<0.001	11.73 (4.83–28.47)
Developmental, psychiatric and behavioral	<0.001	4.22 (2.99–5.95)	<0.001	3.57 (2.51–5.10)
Genitourinary and puberty related	<0.001	5.93 (3.92–9.69)	<0.001	6.60 (3.97–10.97)
Endocrine disorders and diabetes	<0.001	4.38 (2.65–7.22)	<0.001	4.26 (2.55–7.10)
Musculoskeletal and orthopedics	<0.001	5.14 (3.35–7.88)	<0.001	6.57 (4.20–10.27)
Eye conditions	<0.001	6.17 (3.60–10.57)	<0.001	8.77 (4.96–15.51)
Preventive services and vaccinations	0.02	0.61 (0.40–0.92)	0.001	0.45 (0.29–0.71)
Unspecified (e.g., general fatigue)	0.31	1.41 (0.71–2.79)	0.01	2.49 (1.22–5.04)
Routine check-up	Ref			

## Data Availability

The data presented in this study are available on request from the corresponding author due to restrictions related to institutional policies; access requires prior approval from the Institutional Review Board.

## Abbreviations

The following abbreviations are used in this manuscript:

PHC	Primary Healthcare
KSA	Kingdom of Saudi Arabia
OR	Odds ratio
CI	Confidence Interval
VIF	Variance Inflation Factor
